# Long-Term Clinical Outcome of Abdomino-Thoracic Lymphatic Interventions of Traumatic and Non-Traumatic Lymphatic Leakage in Adults

**DOI:** 10.3390/biomedicines11092556

**Published:** 2023-09-18

**Authors:** Lea C. Kaminski, Julia Wagenpfeil, Jens Buermann, Philipp L. Lutz, Julian A. Luetkens, Ulrike I. Attenberger, Christian P. Strassburg, Jörg C. Kalff, Hans H. Schild, Claus C. Pieper

**Affiliations:** 1 Department of Diagnostic and Interventional Radiology, University Hospital of Bonn, 53127 Bonn, Germanyhans.schild@ukbonn.de (H.H.S.); 2 Department of General, Visceral, Thoracic and Vascular Surgery, University Hospital of Bonn, 53127 Bonn, Germany; 3 Department of Internal Medicine I, University Hospital of Bonn, 53127 Bonn, Germany

**Keywords:** chylothorax, chylous ascites, lymphangiography

## Abstract

The aim of this study was to retrospectively evaluate the long-term results of lymphatic interventions in adults with abdomino-thoracic lymphatic pathologies. Management of abdomino-thoracic chylous effusions in adults undergoing X-ray-lymphangiography with or without lymph-vessel embolization (LVE) from 2010–2018 was reviewed. Patients underwent lymphangiography alone when imaging showed normal findings or lymphatic obstruction without leakage or reflux; otherwise, LVE was performed (leakage, reflux, obstruction with leakage or reflux, lymphatic masses). Technical and clinical success, complications, and long-term outcomes were assessed. 78 patients (47 male, median age 56.3 years) were treated for chylous effusions (60.3% traumatic, 39.7% non-traumatic). Lymphangiography showed leakage (48.7%), reflux (14.1%), obstruction (28.2%), lymphatic masses (5.1%), and normal findings (3.8%). Embolization was performed in 49/78 (62.8%) cases. Overall, treatment was clinically successful in 74.4% (mean follow-up of 28 months), with significant differences between LVE and lymphangiography (91.8% vs. 44.8%; *p* < 0.001), traumatic and non-traumatic etiologies (89.4% vs. 51.6%; *p* < 0.001), and leakage locations (*p* = 0.003). The clinical success of LVE did not differ between leakage etiologies or locations. Complications occurred in 5 patients (2/5 needed treatment). Patients survived significantly longer after successful treatment (2679 vs. 927 days; *p* = 0.044) and without malignancy (3214 vs. 1550 days; *p* = 0.043). Lymphatic interventions are safe and effective. LVE should be attempted whenever feasible, as success is high (>90%). Successful intervention has a positive effect on patient survival.

## 1. Introduction

Lymphatic leakages with chylous effusions (chylothorax, chylous ascites, chylopericardium, and cutaneous fistulas) are rare but difficult to treat and associated with considerable morbidity and mortality [1]. Traumatic or iatrogenic causes are the most frequent (e.g., after esophagectomy) [2,3]. Non-traumatic chylous leakages have a broader etiological spectrum, ranging from lymphatic malformations and anomalies to neoplastic and infectious causes [4,5].

When conservative treatment [2,6,7,8,9] with dietary measures fails, surgical treatment is traditionally attempted (e.g., thoracic duct [TD]-ligation) [2,10,11,12,13], but is associated with significant morbidity and mortality in the often multi-morbid patients (14–39% and 1–25%, respectively) [2,14,15,16,17]. Interventional-radiological treatment options such as oil-based X-ray lymphangiography (XRL) or lymph vessel embolization (LVE) are viable alternatives, but results may vary with the type of intervention, leakage location, and etiology [1,2,5,6,18,19,20]. Furthermore, little is known about the long-term results.

Therefore, we conducted a retrospective study in patients with abdomino-thoracic lymphatic leakage, analyzing the treatment outcome with regard to anatomic location and the etiology of leakage.

## 2. Materials and Methods

### 2.1. Patient Cohort

Retrospective data analysis was approved by the institutional review board with a waiver for informed patient consent. The medical records of consecutive adult patients undergoing interventional treatment for abdomino-thoracic lymphatic leakage between 2010 and 2018 were reviewed.

The inclusion criteria were:

- adult age ≥ 18 years,
- presence of clinically confirmed chylous effusion refractory to conservative treatment,
- clinical confirmation of chylous fluid by laboratory investigation (triglyceride levels > 110 mg/dL) and exclusion of a pseudo-chylous effusion (cholesterol levels < 50 mg/dL; ratio cholesterol: triglycerides < 1) [9]
- XRL with or without transabdominal LVE.

Patients with peripheral leakages or other types of treatment (e.g., sclerotherapy) were excluded.

### 2.2. Interventional Technique

All interventions were performed by the same interventional radiologists (C.C.P. and/or H.H.S. with over 10 and 40 years of interventional experience). The techniques employed for XRL and LVE have been described in detail elsewhere [2,14,19,21].

In short, nodal XRL was performed through a bilateral inguinal nodal access (one needle per groin) with an injection of up to 20 mL of iodized oil (Lipiodol^®^, Guerbet, Villepinte, France). No pedal XRL procedures were performed in this cohort. When indicated (see below), catheter-based LVE was performed: a 22G-fine-needle (Cook Medical, Bloomington, IN, USA) was used for fluoroscopy-guided lymph-vessel puncture, and a 2.7F micro-catheter (Renegade^®^, Boston Scientific, Marlborough, MA, USA) was introduced over a guide wire (Transend^®^, Boston Scientific, Marlborough, MA, USA). Target lymphatic vessels for puncture were selected according to individual imaging findings (for thoracic duct embolization, typically the cisterna chyli or the lower part of the thoracic duct). Embolization of the target lymph vessel was performed with coils and/or liquid embolics (Histoacryl^®^, Braun, Melsungen, Germany) mixed with iodized oil (mixture ratio 1:1–1:5) [22,23]. Coils were used in the embolization of large lymph vessels (in most cases, the thoracic duct) in order to prevent the migration of glue (for example, towards the lymphatic venous junction). Glue-only embolization was performed in more peripheral lymphatics without the risk of central glue embolization or lymph node embolization. If catheter-based embolization was not feasible, interstitial lymph node embolization was performed. Thereafter, MCT diet/parenteral nutrition was prescribed for 2–3 days.

### 2.3. Imaging Findings

Intra-interventional XRL findings were categorized by the interventionalists as:

- Normal findings (no lymphatic leakage, reflux, obstruction, or mass)
- Localized lymphatic leakage (i.e., extravasation of contrast medium)
- Chylo-lymphatic reflux (i.e., retrograde flow away from the TD)
- Obstruction of central lymphatic run-off (with or without alternate lymphatic pathways)
- Mass-forming lymphatic malformations.

### 2.4. Treatment Strategy

**Figure 1:**
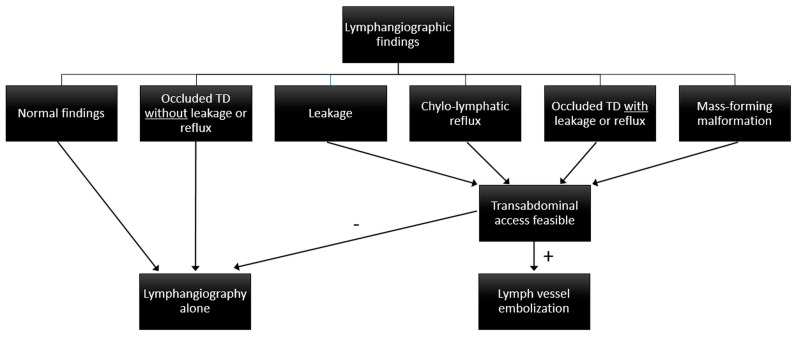
Treatment algorithm for abdomino-thoracic lymphatic leakages.

The interventional strategy depended on the location of leakage and lymphangiographic findings (Figure 1):

- XRL-only was done when normal findings, lymphatic obstruction without leakage or reflux, or no accessible lymph vessel or node were seen.
- LVE was done when lymphatic leakage, reflux, lymphatic obstruction with leakage or reflux, or lymphatic malformations were identified.
- Chylous ascites was a contraindication for central embolization if no pathology of abdominal lymphatics was identified, as this might impair lymphatic run-off and worsen ascites formation.

### 2.5. Data Analysis and Definitions

Data were gathered from patient charts, radiological databases, and picture archiving systems. Based on patient history and imaging findings, the etiology of lymphatic leakage was categorized as traumatic or non-traumatic.

XRL was technically successful when central lymph vessels were opacified. LVE was technically successful when lymph vessels and nodes could be accessed and embolized. Clinical success was defined as the resolution of lymphatic drainage after the intervention without the need for further treatment. Adverse events were rated according to the Common Terminology Criteria for Adverse Events (CTCAE, Version 5).

### 2.6. Statistical Analysis

Analyses were performed using SPSS, V27.0 (IBM, NY, USA). Drainage volumes were compared between leakage locations (abdominal/thoracic/combined), etiology (traumatic/non-traumatic), intervention-type (XRL/LVE), and clinically successful or unsuccessful cases using the Mann-Whitney U-test and the Kruskal-Wallis-test. Imaging findings were compared between leakage locations using the Chi-square test. A comparison of therapeutic success was performed using Fisher’s exact test and. Chi-square tests. Survival analysis was performed using the Kaplan-Meier method with log-rank tests. *p*-values of <0.05 were considered statistically significant.

## 3. Results

### 3.1. Patients

78 patients with chylo-lymphatic leakages (47 male, median age 56.3 years) were included:

- 48/78 patients (61.5%) had thoracic chylous effusions [chylothorax (n = 44), chylopericardium (n = 4)],
- 22/78 (28.2%) had chylous ascites,
- and 8/78 (10.3%) had a combination thereof.

47 patients (60.3%) had a traumatic, and 31 patients (39.7%) had a non-traumatic etiology. Detailed patient characteristics are given in Table 1.

All patients had undergone unsuccessful conservative treatment (median duration: 4 [range 2–52] weeks):

- MCT diet (n = 21),
- parenteral nutrition (n = 23) or
- a sequential combination of both (n = 34) and
- additional octreotide therapy (n = 11).

68/78 patients had an indwelling drainage catheter with median drainage volumes of 1000 mL/day (range 250–8000 mL). In 63/68 patients (92.6%), drainage volume was above 500 mL/day. Daily drainage volumes did not differ significantly between etiologies, leakage locations, or types of interventions (Table 1).

### 3.2. Imaging Findings

**Figure 2:**
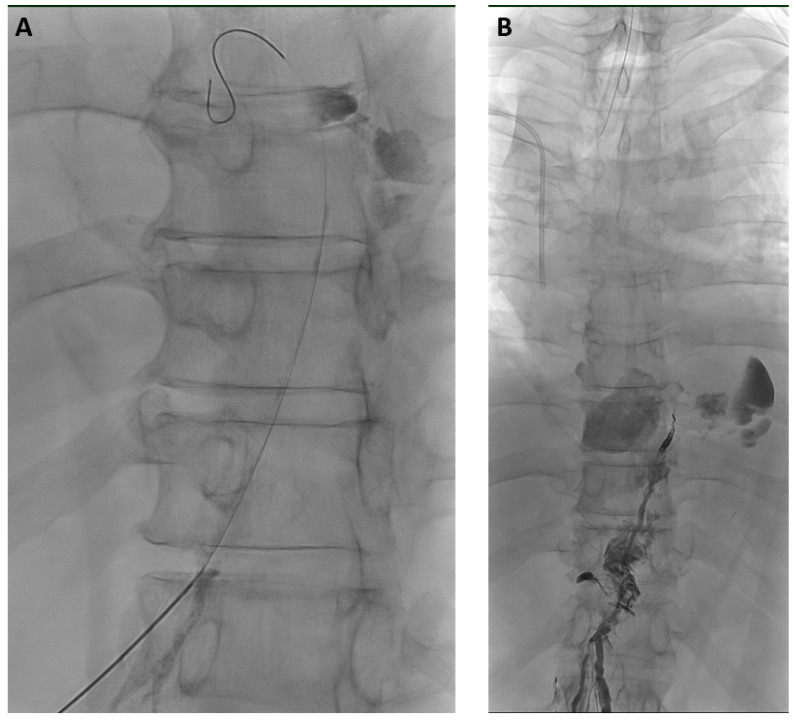
71-year-old man with bilateral chylothorax after esophagectomy. (**A**) X-ray lymphangiography demonstrated transection of the thoracic duct in the lower thorax with active leakage of contrast agent. After puncture of the thoracic duct, the inserted micro-wire already exits the thoracic duct at the leakage-site. (**B**) After thoracic duct embolization with micro-coils and a mixture of NBCA/iodized oil (ratio 1:2) leakage ceased immediately without recurrence or clinical sequelae over a follow-up time of 2.5 years.

**Figure 3:**
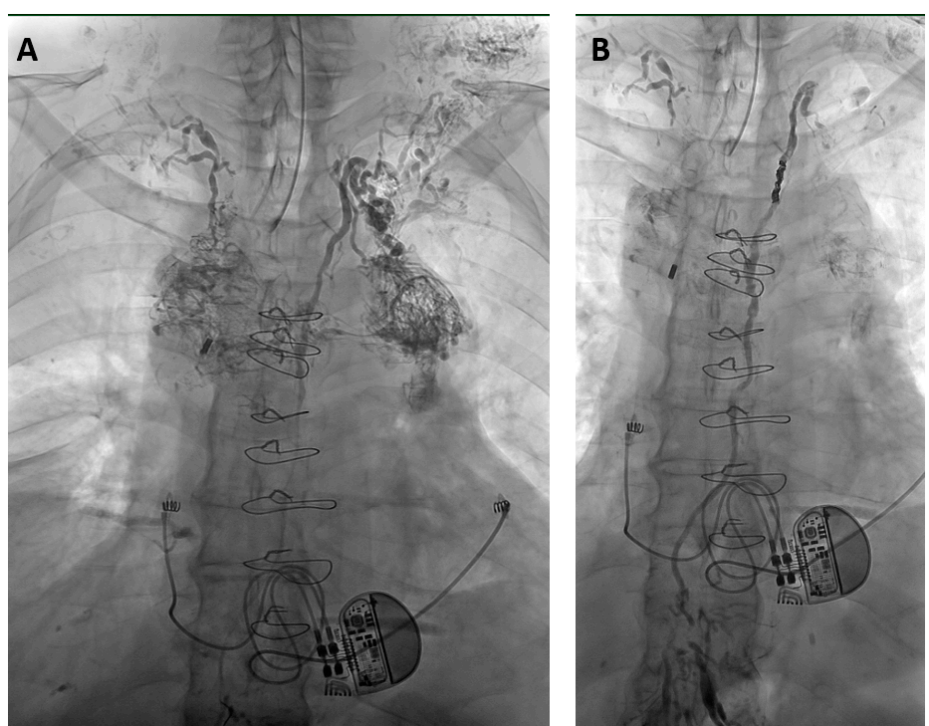
65-year-old man with a non-traumatic bilateral chylothorax and chylopericardium. (**A**) X-ray lymphangiography shows chylous reflux from the upper thoracic part of the thoracic duct into dilated and tortuous lymphatic vessels in the mediastinum and cervical soft tissue. The right-sided lymphatic duct at the right venous angle can also be seen. (**B**) After thoracic duct embolization with micro-coils and a mixture of NBCA/iodized oil (ratio 1:3) leakage ceased within 2 days without clinical sequelae over a follow-up time of 4 years.

XRL was technically successful in all cases. The median amount of iodized oil was 12 mL (range 8–20 mL) for LVE, while 20 mL was administered for XRL-only in all cases. Lymphangiograms demonstrated the cause of effusions in 96.2% (75/78) (Table 2, Figure 2 and Figure 3).

Imaging findings differed significantly between leakage locations (*p* < 0.001). In patients with thoracic effusions, leakage was the most frequent finding (60.4%; 29/48), while with chylous ascites and combined abdomino-thoracic effusions, leakage was observed in only 36.4% (8/22) and 12.5% (1/8) of cases, respectively. Conversely, lymph-vessel obstruction was responsible for chylous ascites in 50% of cases (11/22), while it was present in only 12.5% (6/48) of cases with thoracic effusions.

### 3.3. Interventional Procedures

Based on imaging findings and clinical presentation, XRL-only was performed in 29/78 patients (37.2%). In 21/29 cases, this was done because of a normal lymphangiogram or an obstructive flow pattern without reflux or leakage; in 8/29 cases, XRL alone was done because no access for LVE was identified.

LVE was attempted in 49/78 (62.8%) patients and was technically successful in all cases. Coils and glue were used for LVE in 42/49 (85.7%), while only glue was used for 7 LVEs (14.3%; 2 TD-embolizations, 5 interstitial embolizations).

### 3.4. Clinical Success

**Figure 4:**
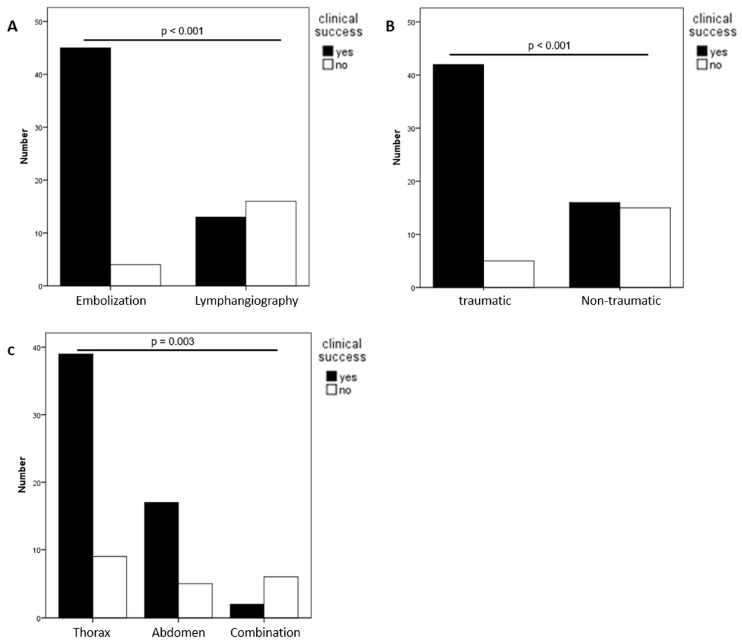
Clinical success of interventional treatment categorized according to treatment type (**A**), etiology (**B**), and leakage location (**C**).

Treatment was clinically successful in 58/78 patients (74.4%). Pre-interventional daily drainage volumes did not differ significantly between successful and unsuccessful cases. Clinical success differed significantly between LVE (45/49, 91.8%) and XRL-only (13/29, 44.8%) (*p* < 0.001) (Table 3, Figure 4A). 12/21 patients (57.1%) in whom XRL-only was performed as planned responded to lymphangiography, while only 1/8 patients (12.5%) in whom LVE would have been the treatment of choice responded to XRL-only (*p* = 0.04).

Clinical success was higher for traumatic compared to non-traumatic causes (89.4% [42/47] vs. 51.6% [16/31], *p* < 0.001) (Figure 4B), which was due to differences in success rates for XRL-only. While LVE was successful in 33/35 traumatic (94.3%) and 12/14 non-traumatic cases (85.7%) (*p* = 0.332), XRL-only was successful in 9/12 traumatic (75.0%) and 4/17 non-traumatic cases (23.5%) (*p* = 0.006).

Concerning leakage locations, treatment was clinically successful in 81.3% (39/48) of thoracic and 77.3% (17/22) of abdominal pathologies, but only in 25% (2/8) of combined abdomino-thoracic effusions (*p* = 0.003) (Figure 4C). Again, this was due to inferior results of XRL-only for thoracic (2/8, 25%) and combined effusions (0/5, 0%), compared to abdominal effusions (11/16, 68.8%) (*p* = 0.011). There was no significant difference in results of embolization in different locations (thoracic: 92.5% (37/40), combined: 66.7% (2/3) and abdominal: 100% (6/6); *p* = 0.213).

### 3.5. Complications

No complications associated with lymphangiography alone were recorded. Complications were observed after LVE in 5 patients, with two being major (2.6%) and three minor (3.8%) (Table 4).

### 3.6. Clinical Course and Survival

Patients were followed over a mean of 863 {40–4036} days. In patients who were successfully treated, neither leakage recurrences nor new leakages were observed during this follow-up period.

**Figure 5:**
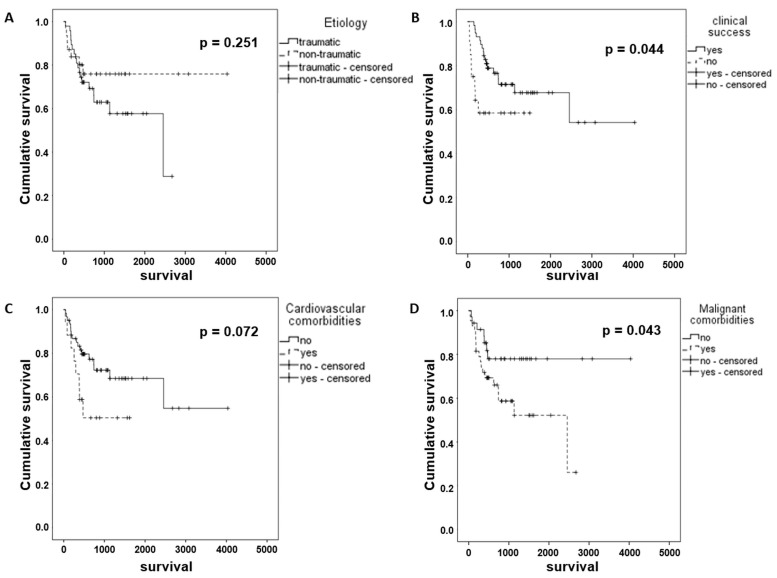
Kaplan-Meier charts demonstrating overall survival comparison for etiology of leakage (**A**), clinical success of interventional treatment (**B**), concomitant cardiovascular (**C**) and malignant disease (**D**).

There were no 30-day fatalities. At the end of follow-up, 53/78 patients (68%) were alive and well. The mean overall survival time was 2536 days and was significantly longer in patients in whom lymphatic leakage was successfully treated (2679 vs. 927 day; *p* = 0.044). Patients with malignant comorbidities had a shorter overall survival than those without malignancy (1550 vs. 3214 days; *p* = 0.043). All other evaluated factors did not show a significant impact on survival (Table 5 and Figure 5A–D).

## 4. Discussion

Over the last decade, interventional radiological treatment of chylo-lymphatic effusions has been shown to be a viable alternative to surgery [9,14,19,20]. Already, XRL alone can be therapeutic. This effect is attributed to the blockage of small, leaking lymph vessels and local sterile inflammation. However, this process is unpredictable and takes time to set in (2–31 days) [6,24]. Hence, considerable variation in success rates of XRL-only have been reported between 7 and 100% [2,6]. In contrast, direct LVE of pathologic or leaking lymph vessels is more predictable and has higher success rates of 70–100% [2]. However, data comparing XRL-only and LVE prospectively are not available to date.

There are currently two different interventional treatment strategies [2]:

1. XRL-only as first-line treatment, with LVE as second-line intervention in cases unresponsive to XRL-only,
2. XRL with planned LVE—if indicated and feasible—in the same intervention.

The data presented here strongly suggest that LVE is the more effective interventional treatment when anatomically feasible (i.e., pathology accessible for catheter-based and/or lymph node embolization) and indicated. XRL-only was clinically successful in 45% of cases, while LVE yielded success in 92% of cases. In our practice, XRL and LVE are generally performed in the same session. Possible advantages of such a “same session” approach include a shorter hospitalization time (patients have often already suffered for many weeks or months from leakage) and lower volumes of iodized oil. Our data show the feasibility of this approach. A drawback of a “same session” strategy, however, is the slightly higher complication rate of LVE (7% in literature) compared to XRL (<5% in literature) [2]. In our cohort, no complications associated with XRL were observed, while 6.5% experienced complications after LVE. However, specific treatment was required for only two patients (2.6%). It is furthermore arguable that XRL-only might already have been effective in some of the patients undergoing LVE. In this respect, it is interesting that XRL-only was successful in only 1/8 of patients in whom transabdominal LVE was intended but was anatomically impossible. Other access routes, like retrograde TD-access [2,25,26], would potentially also have been an alternative for these patients. However, these techniques had not been systematically explored at the time of treatment.

A meta-analysis of interventional treatment of chylothorax including over 400 patients reported a clinical success rate of LVE in more than 80% (92% in traumatic cases) and a success rate of XRL-only in about 50% of traumatic cases [18]. This is in line with the results in our cohort, in which LVE was clinically successful in 93% of traumatic chylothoraces. XRL-only was only employed in two patients with traumatic chylothorax and was clinically successful in one of them.

Non-traumatic chylothoraces are more difficult to treat [18,27]. In the largest single-center report, interventional treatment was only successful in 18/34 (53%): 16/24 (67%) with LVE, but only 2/10 (20%) with XRL-only [27]. Our experiences yielded comparable results, with an overall clinical success rate of 12/18 (67%). As patients benefitted in 11/12 cases from LVE (92%), but only in 1/6 cases from XRL-only (16%), LVE should be attempted when feasible. Although the clinical success of interventional treatment of non-traumatic chylothorax is inferior to that of traumatic chylothorax, surgery has an even poorer clinical success rate of only 27% [28]. Interventional treatment is therefore preferable for non-traumatic chylothoraces. In cases with relevant central lymphatic flow obstruction without leakage or reflux, surgical [29,30] or interventional TD reconstruction [31] should be evaluated, as LVE or ligation may lead to further deterioration in such cases [32].

Although detection of abdominal lymphatic pathologies is more difficult compared to thoracic pathologies (in literature: 55–75% [12,33], in our cohort: 86%), treatment may still be successful. In our cohort, 77% (17/22) benefitted from interventional treatment: all LVEs (6/6) and 11/16 XRL-only led to resolution of chylous ascites. In patients with traumatic chylous ascites, XRL-only leads to ascites resolution in 8/10 (80%) of patients. This is in line with another recently published report [34].

Combined abdomino-thoracic lymphatic leakages are the most difficult to treat. Clinical success was only 25% (2/8). In these cases, LVE impairing central lymphatic run-off can lead to clinical deterioration and is contraindicated [9,32,35]. Biochemical analysis or non-invasive MR-based fat-quantification of fluid from all affected cavities is therefore essential to identify these patients prior to intervention [36].

Long-term follow-up after lymphatic interventions is sparse. Our patients were followed up over a mean time of 2.5 years (longest follow-up: 11 years). Although leg swelling and diarrhea have been reported on long-term follow-up as potential complications in 7–8% [37,38], we did not observe these problems. Furthermore, we did not observe any leakage recurrences after successful interventional treatment. So once an intervention is clinically successful, the beneficial effect seems to be long-lasting. In contrast, uncontrolled chylous effusions have a dismal prognosis, with a mortality rate of up to 50% [10,39]. However, to our knowledge, the impact of successful interventional treatment on patient survival has not been demonstrated. In our patient cohort, the mean overall survival was nearly seven years. Survival was significantly longer after successful treatment of chylous effusions compared to patients with continued leakage (2679 vs. 927 days). As expected, patients with malignant comorbidities had a shorter overall survival (1550 vs. 3214 days).

There are limitations to this study: First, clinical data were analyzed retrospectively with inherent methodological limitations. Second, the diagnosis of the etiology and location of lymphatic pathologies was solely based on clinical presentation and imaging findings, as there are currently no other reliable and available diagnostic tools in clinical routine (such as genetic diagnosis in non-traumatic cases). It is therefore arguable that the imaging-based diagnosis involves assumptions made by the interventionalists during the treatment. However, the diagnosis was performed according to the same algorithm as described above in the entire cohort. The authors acknowledge that further research into multi-parameter diagnostics for lymphatic diseases is necessary. Furthermore, although all therapies followed the algorithm described above, treatment strategies were always tailored to the individual needs of the patient and may also have been influenced by the learning curve of the interventionalists. Therefore, the extent of embolization or iodized oil volume for XRL was not strictly defined. Third, although the patient cohort is one of the largest published so far, the number of patients, especially in the subgroups, is limited, as refractory chylous leakages are generally rare. There are interactions between treatment strategy, etiology, and leakage location that may influence clinical success. However, we did not perform a multi-variate analysis of these parameters owing to the small size of several subgroups. Fourth, we did not perform a randomized comparison between LVE and XRL-only, as embolization was the primary intended mode of treatment. The possible clinical success rate of lymphangiography in those cases with embolization cannot therefore be assessed. However, in cases in which embolization was not feasible or indicated, roughly half of the patients still benefitted from XRL-only, which is in line with previous reports on the clinical efficacy of lymphangiography in the treatment of lymphatic leakages. Concerning the impact of successful treatment of lymphatic leakage on patient survival, it has to be kept in mind that a large proportion of patients had an underlying malignant disease, which could also be shown to have a significant impact on patient survival. Although the data suggests a beneficial influence of successful treatment of lymphatic leakage on survival, further multivariate analysis in larger cohorts is warranted.

## 5. Conclusions

In conclusion, lymph vessel embolization should be attempted whenever feasible, as clinical success is achieved in >90% of patients regardless of the etiology or location of lymphatic leakage. XRL-only is clinically successful in around 50% of cases; results differ considerably between leakage etiologies and locations, with a high treatment success rate in traumatic chylous ascites. Successful interventional treatment leads to a sustained therapeutic effect without the recurrence of leakage and has a positive effect on patient survival.

## Figures and Tables

**Table 1:** Patient characteristics. Characteristics of the entire patient cohort as well as patient subgroups with thoracic (chylothorax/chylopericardium), abdominal (chylous ascites) or abdomino-thoracic (chylothorax/chylopericardium and chylous ascites) effusions. LAM: lymphangioleiomyomatosis.

Parameter	Overall	Thoracic	Abdominal	Abdomino-Thoracic
Number of patients (percentage)	78	48 (61.5%)	22 (28.2%)	8 (10.3%)
Male:Female	47:31	27:21	14:8	6:2
Median age (range)	56.3 (18–86) years	59.4 (18–86) years	64.2 (29–80) years	53.0 (19–61) years
Median daily drainage volume (range)	1000 mL (250–8000 mL)	1000 mL (285–8000 mL)	1000 mL (250–3500 mL)	1850 mL (315–2250 mL)
**Indication for lymphatic intervention**				
Thoracic (chylothorax/chylopericardium)	48 (61.5%)			
Abdominal (chylous ascites)	22 (28.2%)			
Abdomino-thoracic (Combined chylothorax/chylous ascites)	8 (10.3%)			
**Etiology**				
**Traumatic**	**47 (60.3%)**	**30 (62.5%)**	**16 (72.7%)**	**1 (12.5%)**
Tumor surgery	37	23	13	1
Vascular surgery	3	1	2	0
Heart surgery	3	3	0	0
Other surgery	4	3	1	0
**Non-traumatic**	**31 (39.7%)**	**18 (37.5%)**	**6 (27.3%)**	**7 (87.5%)**
No known underlying disease	16	8	4	4
Lymphoma	6	4	1	1
Syndrome (e.g., LAM)	5	5	0	0
Congestive heart failure	2	1	1	0
Venous obstruction	2	0	0	2
**Comorbidities**				
Cardiovascular disease	17 (21.8%)	10 (20.8%)	6 (27.3%)	1 (12.5%)
Malignancy	49 (62.8%)	31 (64.6%)	15 (68.2%)	3 (37.5%)
Liver cirrhosis	4 (5.1%)	1 (2.1%)	3 (13.6%)	0 (0.0%)

**Table 2:** Imaging findings of X-ray lymphangiograms. Findings of the entire patient cohort as well as patient subgroups with thoracic (chylothorax/chylopericardium), abdominal (chylous ascites) or abdomino-thoracic (chylothorax/chylopericardium and chylous ascites) effusions.

	Overall [n = 78]	Thoracic [n = 48]	Abdominal [n = 22]	Abdomino-Thoracic [n = 8]
**Leakage**	38 (48.7%)	29 (60.4%)	8 (36.4%)	1 (12.5%)
**Chylolymphatic reflux**	11 (14.1%)	10 (20.8%)	0 (0.0%)	1 (12.5%)
**Obstruction with reflux or leakage**	4 (5.1%)	3 (6.3%)	0 (0.0%)	1 (12.5%)
**Obstruction without reflux or leakage**	18 (23.1%)	3 (6.3%)	11 (50.0%)	4 (50.0%)
**Lymphatic mass**	4 (5.1%)	3 (6.3%)	0 (0.0%)	1 (12.5%)
**Normal findings**	3 (3.8%)	0 (0.0%)	3 (13.6%)	0 (0.0%)

**Table 3:** Clinical success. Overall clinical success and stratification by type of intervention (embolization vs. lymphangiography alone), by location (thoracic, abdominal or abdomino-thoracic) as well as etiology (traumatic vs. Non-traumatic); *p* < 0.05 is considered statistically significant.

Location/Etiology	Success Overall	Success Embolization	Success lymphangiography	*p*-Value
**Overall**	58/78 (74.4%)	45/49 (91.8%)	13/29 (44.8%)	**<0.001**
Traumatic	42/47 (89.4%)	33/35 (94.3%)	9/12 (75.0%)	0.062
Non-traumatic	16/31 (51.6%)	12/14 (85.7%)	4/17 (23.5%)	**0.001**
*p*-value (Traumatic vs. Non-traumatic)	**<0.001**	0.332	**0.006**	
**Thoracic**	39/48 (81.3%)	37/40 (92.5%)	2/8 (25.0%)	**<0.001**
Traumatic	27/30 (90.0%)	26/28 (92.9%)	1/2 (50.0%)	0.051
Non-traumatic	12/18 (66.7%)	11/12 (91.7%)	1/6 (16.7%)	0.001
*p*-value (Traumatic vs. Non-traumatic)	**0.045**	0.896	0.346	
**Abdominal**	17/22 (77.3%)	6/6 (100%)	11/16 (68.8%)	0.119
Traumatic	14/16 (87.5%)	6/6 (100%)	8/10 (80.0%)	0.242
Non-traumatic	3/6 (50.0%)	0/0 (0%)	3/6 (50.0%)	NA
*p*-value (Traumatic vs. Non-traumatic)	0.062	NA	0.210	
**Combined**	2/8 (25.0%)	2/3 (66.7%)	0/5 (0.0%)	**0.035**
Traumatic	1/1 (100%)	1/1 (100%)	0/0 (0.0%)	NA
Non-traumatic	1/7 (14.3%)	1/2 (50.0%)	0/5 (0.0%)	0.088
*p*-value (Traumatic vs. Non-traumatic)	0.064	0.386	NA	
*p*-value (Thoracic vs. Abdominal vs. abdomino-thoracic))	**0.003**	0.213	**0.011**	

**Table 4:** Complications of lymph-vessel embolizations.

Complication	No.	Cause	CTCAE Grade	Treatment	Outcome
**Biliary peritonitis**	1	Transgression of gallbladder	4	Cholecystectomy	Further course unremarkable
**Bleeding from branch of left hepatic artery**	1	Mandatory therapeutic anticoagulation due to cardiac-assist-device	3	Transcatheter embolization of bleeding vessel	Further course unremarkable
**Edematous pancreatitis**	1	Transgression of pancreas	2	Parenteral nutrition	Further course unremarkable
**Upper extremity vein thrombosis**	1	unknown	2	Heparinization	Further course unremarkable
**Pulmonary glue migration**	1	Transgression of vein near lymphtic puncture site	1	none	Further course unremarkable

**Table 5:** Patient survival. Results of univariate analysis of patient survival. *p* < 0.05 (Chi-square test) was considered statistically significant.

Parameter	Mean Survival [Days] (95% CI)	Univariate *p*-Value
**Overall**	2536 (2004;3068)	
**Gender**		0.397
Female	1941 (1405;2478)	
Male	2518 (1785;3251)	
**Etiology**		0.251
Traumatic	1675 (1328;2022)	
Non-traumatic	3111 (2510;3713)	
**Location**		0.587
Thorax	2522 (1866;3177)	
Abdomen	1956 (1423;2489)	
Combination	875 (441;1309)	
**Treatment**		0.907
Embolization	2655 (2096;3214)	
Lymphangiography	2081 (1560;2603)	
**Clinical success**		**0.044**
Yes	2679 (2104;3253)	
No	927 (621;1234)	
**Comorbidities**		
Cardiovascular		0.072
Yes	2687 (2110;3264)	
No	952 (614;1290)	
Malignant		**0.043**
Yes	3214 (2672;3756)	
No	1550 (1170;1930)	

## Data Availability

Data is unavailable due to privacy restrictions of data in a clinical treatment context.

## References

[1] Schild H.H., Strassburg C.P., Welz A., Kalff J. (2013). Treatment options in patients with chylothorax. Dtsch. Ärzteblatt Int..

[2] Pieper C.C., Hur S., Sommer C.M., Nadolski G., Maleux G., Kim J., Itkin M. (2019). Back to the Future: Lipiodol in Lymphography-From Diagnostics to Theranostics. Investig. Radiol..

[3] Lee E.W., Shin J.H., Ko H.K., Park J., Kim S.H., Sung K.B. (2014). Lymphangiography to treat postoperative lymphatic leakage: A technical review. Korean J. Radiol..

[4] Nadolski G. (2016). Nontraumatic Chylothorax: Diagnostic Algorithm and Treatment Options. Tech. Vasc. Interv. Radiol..

[5] Goity L.D., Itkin M., Nadolski G. (2020). An Algorithmic Approach to Minimally Invasive Management of Nontraumatic Chylothorax. Semin. Interv. Radiol..

[6] Sommer C.M., Pieper C.C., Itkin M., Nadolski G.J., Hur S., Kim J., Maleux G., Kauczor H.U., Richter G.M. (2020). Conventional Lymphangiography (CL) in the Management of Postoperative Lymphatic Leakage (PLL): A Systematic Review. RöFo-Fortschritte Auf Dem Geb. Der Röntgenstrahlen Und Der Bildgeb. Verfahr..

[7] Alejandre-Lafont E., Krompiec C., Rau W.S., Krombach G.A. (2011). Effectiveness of therapeutic lymphography on lymphatic leakage. Acta Radiol..

[8] Gruber-Rouh T., Naguib N.N.N., Lehnert T., Harth M., Thalhammer A., Beeres M., Tsaur I., Hammersting R., Wichmann J.L., Vogl T.J. (2014). Direct lymphangiography as treatment option of lymphatic leakage: Indications, outcomes and role in patient’s management. Eur. J. Radiol..

[9] Pieper C.C. (2023). Back to the Future II-A Comprehensive Update on the RapidlyEvolving Field of Lymphatic Imaging and Interventions. Investig. Radiol..

[10] McGrath E.E., Blades Z., Anderson P.B. (2010). Chylothorax: Aetiology, diagnosis and therapeutic options. Respir. Med..

[11] Cope C. (1998). Diagnosis and treatment of postoperative chyle leakage via percutaneous transabdominal catheterization of the cisterna chyli: A preliminary study. J. Vasc. Interv. Radiol..

[12] Nadolski G.J., Chauhan N.R., Itkin M. (2018). Lymphangiography and Lymphatic Embolization for the Treatment of Refractory Chylous Ascites. Cardiovasc. Interv. Radiol..

[13] Weniger M., D’Haese J.G., Angele M.K., Kleespies A., Werner J., Hartwig W. (2016). Treatment options for chylous ascites after major abdominal surgery: A systematic review. Am. J. Surg..

[14] Schild H.H., Naehle C.P., Wilhelm K.E., Kuhl C.K., Thomas D., Meyer C., Textor J., Strunk H., Willinek W.A., Pieper C.C. (2015). Lymphatic Interventions for Treatment of Chylothorax. RöFo-Fortschritte Auf Dem Geb. Der Röntgenstrahlen Und Der Bildgeb. Verfahr..

[15] Cerfolio R.J., Allen M.S., Deschamps C., Trastek V.F., Pairolero P.C. (1996). Postoperative chylothorax. J. Thorac. Cardiovasc. Surg..

[16] Reisenauer J.S., Puig C.A., Reisenauer C.J., Allen M.S., Bendel E., Cassivi S.D., Nichols F.C., Shen R.K., Wigle D.A., Blackmon S.H. (2018). Treatment of Postsurgical Chylothorax. Ann. Thorac. Surg..

[17] Kariya S., Nakatani M., Ueno Y., Yoshida A., Ono Y., Maruyama T., Komemushi A., Tanigawa N. (2018). Transvenous retrograde thoracic ductography: Initial experience with 13 consecutive cases. Cardiovasc. Interv. Radiol..

[18] Kim P.H., Tsauo J., Shin J.H. (2018). Lymphatic Interventions for Chylothorax: A Systematic Review and Meta-Analysis. J. Vasc. Interv. Radiol..

[19] Schild H., Hirner A. (2001). Percutaneous translymphatic thoracic duct embolization for treatment of chylothorax. RöFo-Fortschritte Auf Dem Geb. Der Röntgenstrahlen Und Der Bildgeb. Verfahr..

[20] Itkin M., Kucharczuk J.C., Kwak A., Trerotola S.O., Kaiser L.R. (2010). Nonoperative thoracic duct embolization for traumatic thoracic duct leak: Experience in 109 patients. J. Thorac. Cardiovasc. Surg..

[21] Pamarthi V., Stecker M.S., Schenker M.P., Baum R.A., Killoran T.P., Suzuki Han A., O’Horo S.K., Rabkin D.J., Fan C.M. (2014). Thoracic duct embolization and disruption for treatment of chylous effusions: Experience with 105 patients. J. Vasc. Interv. Radiol..

[22] Kuetting D., Schild H.H., Pieper C.C. (2019). In Vitro Evaluation of the Polymerization Properties of N-Butyl Cyanoacrylate/Iodized Oil Mixtures for Lymphatic Interventions. J. Vasc. Interv. Radiol..

[23] Kuetting D., Kupczyk P., Dell T., Luetkens J.A., Meyer C., Attenberger U.I., Pieper C.C. (2022). In Vitro Evaluation of Acrylic Adhesives in Lymphatic Fluids-Influence of Glue Type and Procedural Parameters. Biomedicines.

[24] Nadolski G.J., Itkin M. (2013). Thoracic duct embolization for nontraumatic chylous effusion: Experience in 34 patients. Chest.

[25] Pieper C.C., Schild H.H. (2015). Direct Cervical Puncture for Retrograde Thoracic Duct Embolization in a Postoperative Cervical Lymphatic Fistula. J. Vasc. Interv. Radiol..

[26] Jun H., Hur S. (2020). Interventional Radiology Treatment for Postoperative Chylothorax. Korean J. Thorac. Cardiovasc. Surg..

[27] Nadolski G., Itkin M. (2013). Thoracic duct embolization for the management of chylothoraces. Curr. Opin. Pulm. Med..

[28] Maldonado F., Cartin-Ceba R., Hawkins F.J., Ryu J.H. (2010). Medical and surgical management of chylothorax and associated outcomes. Am. J. Med. Sci..

[29] Lindenblatt N., Gutschow C.A., Vetter D., Puippe G., Broglie Däppen M., Schneiter D., Uyulmaz S., Giovanoli P., Pieper C.C., Grünherz (2022). Lympho-venous anastomosis for the treatment of congenital and acquired lesions of the central lymphtic system: A multidisciplinary treatment approach. Eur. J. Plast. Surg..

[30] McGraw J.R., Itkin M., Kovach S.J. (2023). Lymphangiography-Guided Thoracic Duct Surgical Lymphovenous Bypass for Distal Thoracic Duct Occlusion. J. Vasc. Interv. Radiol..

[31] Smith C.L., Hoffman T.M., Dori Y., Rome J.J. (2020). Decompression of the thoracic duct: A novel transcatheter approach. Catheter. Cardiovasc. Interv..

[32] Pimpalwar S., Chinnadurai P., Chau A., Pereyra M., Ashton D., Masand P., Krishnamurthy R., Jadhav S. (2018). Dynamic contrast enhanced magnetic resonance lymphangiography: Categorization of imaging findings and correlation with patient management. Eur. J. Radiol..

[33] Majdalany B.S., Khayat M., Downing T., Killoran T.P., El-Haddad G., Khaja M.S., Saad W.A. (2018). Lymphatic interventions for isolated, iatrogenic chylous ascites: A multi-institution experience. Eur. J. Radiol..

[34] Verhaeghe L., Holsbeeck A.V., Bonne L., Claus E., Marrannes J., Vandenbulcke R., Jochmans I., Pirenne J., Maleux G. (2023). Therapeutic lymphangiography with ethiodized oil for the management of lymphoceles and chylous ascites. Diagnostic and Interventional Imaging.

[35] Benjamin J., O’Leary C., Hur S., Gurevich A., Klein W.M., Itkin M. (2023). Imaging and Interventions for Lymphatic and Lymphatic-related Disorders. Radiology.

[36] Kuetting D., Luetkens J., Fimmers R., Sprinkart A.M., Attenberger U., Pieper C.C. (2020). MRI Assessment of Chylous and Nonchylous Effusions: Use of Multipoint Dixon Fat Quantification. Radiology.

[37] Milsom J.W., Kron I.L., Rheuban K.S., Rodgers B.M. (1985). Chylothorax: An assessment of current surgical management. J. Thorac. Cardiovasc. Surg..

[38] Laslett D., Trerotola S.O., Itkin M. (2012). Delayed complications following technically successful thoracic duct embolization. J. Vasc. Interv. Radiol..

[39] Shackcloth M.J., Poullis M., Lu J., Page R.D. (2001). Preventing of chylothorax after oesophagectomy by routine pre-operative administration of oral cream. Eur. J. Cardiotharac. Surg.

